# Ultrasound-assisted modification of oat protein isolates: Structural and functional enhancements

**DOI:** 10.1016/j.ultsonch.2024.107204

**Published:** 2024-12-16

**Authors:** Hamad Rafique, Pai Peng, Xinzhong Hu, Kanza Saeed, Muhammad Zubair Khalid, Waseem Khalid, Sonia Morya, Tawfiq Alsulami, Robert Mugabi, Gulzar Ahmad Nayik

**Affiliations:** a College of Food Engineering and Nutritional Science, Shaanxi Normal University, Xi'an, Shaanxi 710119, China; b Faculty of Food Technology and Nutrition Sciences, University of Biological and Applied Sciences, Lahore, 53400, Pakistan; c Department of Food Science, Faculty of Life Sciences, Government College University, Faisalabad, Punjab, Pakistan; d Department of Organic Chemistry, Faculty of Chemical Sciences and Technologies, University of Castilla, La Mancha, 13071 Ciudad Real, Spain; e Department of Molecular Food Chemistry and Food Development, Institute of Food and One Health, Gottfried Wilhelm Leibniz University Hannover, Hannover, Germany; f Department of Food Technology and Nutrition, School of Agriculture, Lovely Professional University, Phagwara, 144411, Punjab, India; g Department of Food Science & Nutrition, College of Food and Agricultural Sciences, King Saud University, Riyadh 11451, Saudi Arabia; h Department of Food Technology and Nutrition, Makerere University, Kampala, Uganda; i Marwadi University Research Centre, Department of Microbiology, Marwadi University, Rajkot, Gujarat 360003, India

**Keywords:** Ultrasound, Oat protein isolate, Fourier transform infra-red spectroscopy (FTIR), Circular dichroism spectrophotometer, Micro-structure

## Abstract

Escalating global protein demand necessitates the commercialization of protein rich products. Oat is a promising high-quality protein source but it requires structural and functional modifications to diversify its application. The current investigation was focused on the impact of different powers of ultrasonic waves (200, 400, and 600 W) on structural and functional characteristics of oat protein isolates to improve its techno-functional properties. Higher strength ultrasound waves generated flat sheet structures which were observed while analyzing microstructure of oat protein isolate (OPI). However, non-significant variation in molecular weight distribution were observed in different treatments. At 600 W power of ultrasonic waves the protein fragments show local accumulation, increased α-helix content. Due to uncoiling of protein structure decrease in β-sheets and β-turns was also observed at 600 W. Protein turbidity decreased significantly under low power ultrasonic treatment (200 W) which significantly increased at higher power. Moderate ultrasonic treatment (400 W) promoted protein dissolution, and maintained a good balance between β-sheets (71.04 ± 0.08), α-helix (16.27 ± 0.02) and β-turns (12.68 ± 0.03), exhibiting optimized flexibility and structural integrity. Whereas, higher strength (600 W) significantly destroyed protein structure. The amino acid content decreased significantly with increasing ultrasonic power. The thermal characteristics of OPI remained unaffected after ultrasound treatment. In conclusion, modifications of secondary and tertiary structure induced by moderate ultrasonic treatment (400 W) improved functional properties of OPI. The 400 W treatment resulted in highest essential amino acid content (EAA) i.e., 22.75 ± 0.82 mg/100 mg and total amino acid content (TAA) i.e., 64.94 ± 2.7 mg/100 mg, which are significantly higher than WHO and FAO standards, suggesting best total and essential amino acid production in comparison to other treatments.

## 1 Introduction

Global population is continuously escalating, making it difficult for conventional protein sources to suffice the ever-increasing demand [1]. In order to overcome this insufficiency, there is a need for new and sustainable dietary protein sources [2]. With popularization of alternative protein source, the market is expected to flourish at an annual rate of 14 % by the end of year 2024. According to National Research Council Canada (NRCC) in year 2022, market of plant-based proteins reached USD 10.8 billion [3]. Research evidences regarding the health benefits of oat (*Avena sativa*) surged its utilization as nutritious food. For gluten and dairy free formulations, oat proteins isolated (60–90 % proteins) offers smooth mouth feel and neutral flavor. To capitalize the currents trends of increasing market demand for plant-based foods, oat protein is well positioned in terms of versatility and sustainability for designing clean-label plant based functional foods [4]. Oat proteins are rich source of essential amino acids (leucine, lysine, threonine, isoleucine, valine, phenylalanine) and non-essential amino acids (glutamic acid alanine and serine)[5]. Oats contain high percentage of soluble fibers, including β-glucan, a prebiotic component that promote serum cholesterol reduction and reduced the incidence of other non-communicable diseases like obesity, type 2 diabetes mellitus and cardiovascular diseases [6]. In addition to nutritional and health benefits, oat protein exhibits functional characteristics like foaming and emulsification properties [7].

The major storage protein in oats is globulin (12 s), it is a 320 kDa hexamer having submits of 54 kDa comprising disulphide bond linked acid chain of 32 kDa and a basic chain of 22 kDa [8]. Depending on the variety the total protein content of oats varies between 11–24.5 % [9]. Techno-functionality and high protein content of oats facilitates the formulation of protein rich food products. However, under slight acidic and neutral pH relative solubility of oat protein is relatively low (40 %) [7]. To overcome this issue of low solubility, novel structural modification strategies can ward off, this techno-functionality constraint of oat protein. In recent years, scientists are focusing on development and application of physical techniques that alter the protein structure without exogenous chemical reagent resorting [9]. Table 1. Shows emerging technologies for oat protein treatment*.* These include atmospheric cold plasma [10], high pressure [11], pulsed electric field [12] and extrusion [13]. Another renown promising technology to modify physical protein structure is ultrasound processing [14].

**Table 1:** Emerging technologies for oat protein treatment.

**Sr.**	**Technique**	**Mechanism of action**	**Advantages**	**Disadvantages**	**References**
1	High-Pressure Processing	Non-covalent bonds affect the structure by enhancing surface hydrophobicity, causing protein denaturation and aggregation	Improve functionality, digestibility inactivation antinutritional factors	Destructive effect on the quaternary and tertiary structure	[15]
2	Ultrasound	Ultrasound generate air bubbles in liquid phase, increase volume and explode, resulting in cavity formation	Quick and cost effective modify globular proteins structure and functional properties	Disrupt complete protein microstructure	[16]
3	Pulsed Electric Field	Across cell membrane PEF induces a critical electrical potential facilitating an easier protein extraction	Increased mass transfer, high extraction yield, less processing time, low compounds degradation	Detrimental impact on textural properties	[12]
4	Ohmic Heating	Electric current pass through food material due to electrolytic components, generating internal heat	Improve the electrical conductivity, enhance permeability, better extraction rates of biomolecules	Cause changes in cell membranes, viscosity, pH, color, and rheology of food	[17]
5	Microwave	Heat is generated by altering the electromagnetic field by cause conformational changes in protein	Inactivate antinutritional factors and improves protein digestibility proteins	Destroy nutrients in foods	[18]
6	Cold Plasma	Gas discharge plasma involves treatment of material for superficial effects (polymers functionalization and food decontamination). Reactive species are produced by ionization resulting in changes on the food surfaces	Cause surface modification, inactivation of deteriorating enzymes, reduction of food allergenicity	Mild oxidation of the proteins	[10]
7	Enzymatic Processes	Enzymes causes plant cell walls disruption and enhance the extraction yield by decoupling proteins attached to the plant polysaccharide matrix	High yield, non-toxic and cost effective	Disrupt protein structure	[19]

Table 1**.** Emerging technologies for oat protein treatment [15], [16], [12], [17], [18], [10], [19].

Ultrasound radiation generate mechanical waves of frequency higher than human hearing threshold. These waves have two major classifications i.e., high frequency-low intensity ultrasound (LIU, 100 kHz-1 MHz, and power < 1 W/cm^2^) and low-frequency-high intensity ultrasound (HIU, 16–100 kHz, and 10–1000 W/cm^2^) [9]. The second type of wave i.e., HIU has the potential to generate extremely high variations in pressure upon periodic transfer of energy through mechanical motion of probe that leads formation of small sized bubbles also known as cavities and the phenomena is regarded as cavitation [20], [21]. Cavitation disrupt compact structure of protein by partial unfolding of protein helix, that positively impact physicochemical and functional characteristics of protein [22]. In recent years, this technology has been applied successfully for treatment of proteins from moving sources including cereal, legume and animal derived proteins, for functionality expansion [23]. Due to improved protein recovery and efficient protein release extraction process is completed within few minutes to hours, without involving the use of toxic chemicals[24]. UAE is an environmentally friendly and healthier extraction process that enhance functional characteristics (solubility, gelation and emulsification)[25] and preserve nutritional quality of oat protein isolates by preventing heat degradation of essential amino acids (lysine, leucine and valine) [26] and bioactive compounds (β-glucans and avenanthramides) [27]. Jahan et al. [28] observed significant improvement in functional properties and 30 % higher protein yield at optimum process parameters (duration, power and duration). Wang et al. [29] conducted a study focused on impact of ultrasonic waves on functional characteristics like emulsification and solubility. The results demonstrated notable improvement in functional properties. Singla et al. [30] investigated energy efficiency of ultrasound treatment in comparison to conventional heating techniques and the results revealed that by using ultrasound waves, better protein yield can be achieved with less energy expenditure. For controlling the protein extraction efficiency, retaining protein functionality, energy consumption optimization and ensuring sustainable production processes. Careful optimization of ultrasound power is required to balance the benefits of better solubility, higher yields and protein quality preservation with potential risks of excessive energy consumption and protein degradation. Thus, in order to achieve best possible outputs of oat protein isolation, ultrasound power is a key parameter [31].

The purpose of the current study was to investigate the impact of different strengths of ultrasound waves (200 W, 400 W and 600 W) on microstructure, secondary structure, tertiary structure, molecular, weight, turbidity, hydrophobicity thermal behavior, and amino acid composition of oat protein isolates. The power level of ultrasound waves was selected to understand effect of mild, moderate and high-power ultrasonic waves on the structural and nutritional characteristics of OPI. The primary goal of this study was to apply ultrasound processing to produce stable and high-quality oat-protein based food products.

## 2 Materials and methods

### 2.1 Procurement of raw material

Oats were purchased from Guilin Seamild Food Co. Ltd., China. Oat composition was 17.90 % proteins, 6.43 %, β-glucan, 49.32 % starch and 8.13 % lipids.

### 2.2 Oat protein isolate (OPI) preparation

OPI was prepared by following the methodology designed by Yue et al. [32] with slight modifications. Grounded defatted oats were dissolved in deionized distilled water, at room temperature and the suspension was stirred for 1 h. The prepared slurry was then centrifuged at 4000 rpm for 20 min. The supernatant was carefully separated after the precipitates were settled. The pH of the sample was adjusted to 4.5 using 1.0 M HCl for protein isolation. To produce oat protein, OPI was freeze-dried for 48 h. The protein content of the OPI was determined using Kjeldahl method. For further testing, powdered oat protein was stored at −20 °C in plastic bags.

### 2.3 Ultrasound-pretreated hydrolysis of OPI

The OPI aqueous suspension (5% w/v OPI) was placed in a soundproof enclosure and subjected to ultrasound treatment at varying power levels (200W, 400W, and 600W) using a 25 kHz probe (Φ6). The samples were treated for 15 minutes at a controlled temperature of 30°C with an ultrasonic apparatus (Xinyi 950-W, Ningbo Xinyi Ultrasonic Instrument Co., Ltd.)

### 2.4 Micro-structure analysis

A scanning electron microscope (SEM) (SU8220, Hitachi High-Technologies Corporation, Japan) at 15 kV acceleration voltage was used to study morphological characteristics of oat protein isolate as per the method designed by Lan et al. [33].

### 2.5 Sodium dodecyl sulphate–polyacrylamide gel electrophoresis (SDS- PAGE) of OPI

The molecular weight distribution of both control and ultrasound-treated samples was evaluated using the SDS-PAGE technique, following the protocol described by Zha et al. [34] using SDS-PAGE technique. A mixture of 12 % separating gel and a 4 % stacking gel were prepared, and a 5 mg/mL sample was dissolved with 2x loading buffer. The mixture was electrophoresed at 30A for 3 h. Upon completing gel electrophoresis, a staining fixation solution was prepared with Coomassie Brilliant Blue R-250, isopropanol, and acetic acid and used to stain the gel for two hours. The gel was then distained by washing it 3–5 times.

#### 2.5.1 Amino acid composition of OPI

An acid hydrolysis technique (GB/T5009. 124–2016) was used on amino acid analyzer (HITACHIL-8900, Hitachi, Tokyo, Japan) to determine amino acid composition of OPI. A 100 mg sample of powdered OPI was digested in a digestion tube with 6 M hydrochloric acid (5 mL and 99.99 % purity). The nitrogen gas was introduced after ultra-sonication for 2 min, and the tube was sealed air tight. The sealed samples were then subjected to microwave digestion (180 °C for 30 min). Once digestion was complete, the tubes were cooled to room temperature and removed from the setup. Following thorough mixing, 50 mL of distilled water was added to the solution.1 mL portion of the filtrate was combined with 1 mL of 0.5 M NaOH. 10 mL of distilled water was then added to the mixture and filtered by passing through 0.45 µm inorganic filter membrane. The prepared samples were analyzed using amino acid analyzer (0.1 mL/min). The separation column and the reaction column were maintained at 57 °C and 135 °C, respectively. The result of each sample was recorded in triplicate for accuracy [35].

### 2.6 Fourier transform infra-red spectroscopy (FTIR) analysis of OPI

Secondary structure of OPI was determined at room temperature using FTIR spectrophotometer (vertex-70, Braker, Germany) as described protocol by Peng et al. [36]. A ratio of 1:100 was used as mixture of sample and potassium bromide, respectively, dried at 48 °C for 12 h. The dried mixture was then finely grounded using mechanical grinder (HSM 3000, Huanlong Machinery, China). A 20 mg portion of the powder was compressed into thin slices using a force of 11 lbs. The scanning process used a background resolution of 4 cm^−1^, with 64 scans done for each test over a range of 4000 cm^−1^ to 400 cm^−1^. To ensure accuracy each sample result was analyzed in triplicate.

For quantification of different secondary structures of OPI, the amide I (1700–1600 cm^−1^) band in FT-IR was analyzed using Peakfit (version 4.12, SPSS Inc., Chicago, IL, USA) and Omnic (version 8.0, Thermo Nicolet Inc., Waltham, MA, USA). The amide I band was assigned values as: β-sheets (1635–1625 cm^−1^), intermolecular β-sheets (1618–1612 cm^−1^), parallel β sheets (1695–1685 cm^−1^), random coil (1655–1640 cm^−1^), β-turn (1680–1670 cm^-l^) and α-helices (1665–1658 cm^−1^).

### 2.7 Circular dichroism analysis of OPI (CD)

Circular dichroism spectrophotometer (Jasco J-1000, JASCO, Japan) was used to analyze the tertiary structure of OPI by following the method designed by Yue et al. [32]. 0.2 g OPI was added to 1 mL of phosphate buffer solution (pH 7.0, strength 10 mm). The spectrophotometric readings were recorded at 260 nm.

### 2.8 Thermogravimetric analysis (TGA)

Thermogravimetric analysis (TGA) was performed using a thermal analysis system (Q1000 TA Instruments, New Castle, USA). A 5 g sample was heated at a rate of 10 °C per minute across a temperature range from 30-120 °C. In cooling system liquid nitrogen was circulated thoroughly at a flow rate of 20 mL/min [37].

### 2.9 Statistical analysis

The statistical analysis was conducted using the Statplus software. All experiments were analyzed in triplicate for accuracy. Analysis of variance (ANOVA) was applied based on a completely randomized design (CRD), with a significance level set at *p* < 0.05. Tukey’s significant difference (HSD) was used for multiple pairwise comparison.

## 3 Results and discussion

### 3.1 Micro-structure of different oat protein isolates

**Fig. 1:**
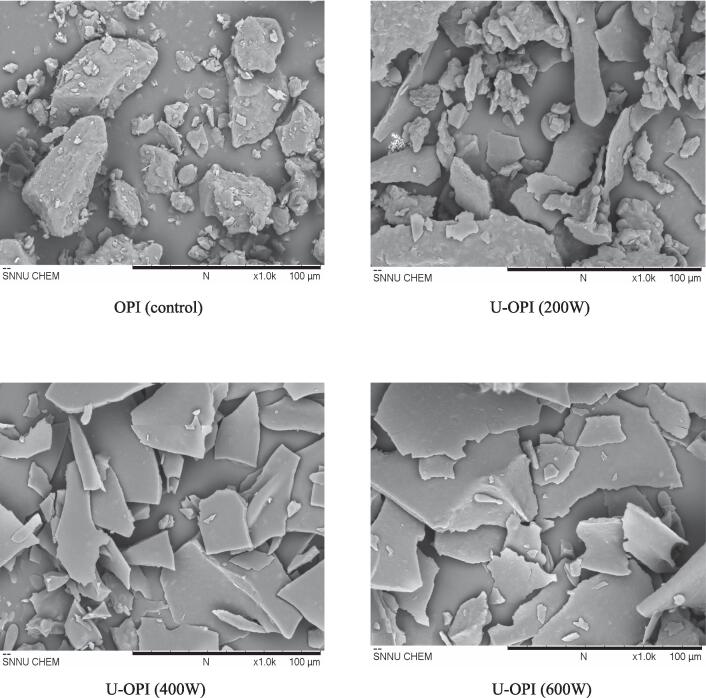
Impact of ultrasound-treatment on micro-structure of oat protein isolates (SEM).

Fig. 1 illustrates the microstructural variations in oat protein isolates (OPI) subjected to different ultra-sonic wave treatments. In the control sample the OPI exhibited a blocky and relatively dense structure. While, in the ultrasonic treated sample at 200 W, the protein clusters disintegrated to form smaller and more dispersed particles. However, the samples treated at higher ultrasonic intensities (400 W and 600 W), showed significant changes in the protein structure as flattening into sheet-like structural formations.

### 3.2 Molecular weight distribution of oat protein isolates

**Fig. 2:**
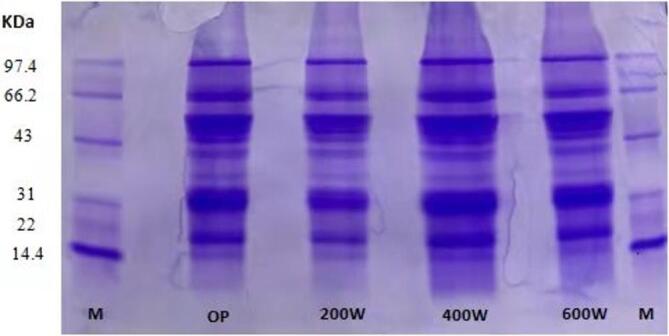
Impact of ultrasound-treatment on molecular weight distribution of oat protein isolates.

The molecular weight distribution of proteins remained largely unchanged in terms of insignificant difference across different ultrasonic treated samples (Fig. 2). But, at 400 W and 600 W, the intensity of the smaller molecular weight bands increased, suggesting that these ultrasonic power levels led to the breakdown or depolymerization of protein molecules. This phenomenon appears to be closely related with modifications in the secondary and higher-order structures of the proteins [38].

Ultrasonication generate mechanical shear force along with cavitation effect that disrupt the protein molecule structural integrity. A recent investigation reported similar outcomes where exposure to ultrasound waves lead to protein fragmentation, thus, altering the molecular conformation [39]. Kamal et al. [40] reported that peptide bonds are cleaved by ultrasonic cavitation resulting in generation of smaller protein fragments. Our study well aligns with these outcomes indicating ultrasonic power facilitate protein depolarization, unfolds protein molecule and expose hydrophobic regions. This structural disruption makes them more susceptible to cleavage, resulting in improved functional properties [41].

### 3.3 Secondary structure of oat protein isolates

FT-IR spectroscopy was used to examine the effects of ultrasound waves on the secondary structure of OPI. During the analysis for characterization of OPI secondary structure, it was observed that the amide I spectral bands primarily attributed to the stretching of the C-O bonds in the protein molecules, which are sensitive to variation in the protein's secondary structure [42]. Amide I corresponding peak regions confirms the presence of random coil structures, a-helix, β-sheet and β-turn [43].

Structure of α-helix exhibit sequenced and stable conformation while the β-sheets of protein shows zigzag structural conformation. A comparison of the α-helix and β-sheet structures shown that β-sheets exhibited a higher degree of unfolding. When heat is applied to proteins, the α-helix structure transforms into β-sheets [36]. Table 2. shows the impact of ultrasound waves on β-sheets, α-helix and β-turn, significant variation was observed in protein secondary structure with increase in power of ultrasonic waves. Contraction and aggregation of proteins occurred when ultrasonic treatment at 200 W. It is also shown that the ultrasonic treatment at 400 W exhibited a relative decrease in α-helix content while the β-sheet content increased, resulting in expansion of secondary structure of protein due to its microstructure breakdown. When 600 W ultrasonic wave power was used on OPI, protein fragments accumulated locally, resulting in escalated α-helix content and β-sheet content decline.

**Table 2:** Impact of different powers of ultrasound-treatment on Secondary structure of oat protein isolates (FTIR).

**Samples**	**β-Sheets**	**α-Helix**	**β-Turn**
OPI	74.01^a^ ± 0.14	15.04^c^ ± 0.04	10.93^d^ ± 0.09
U-OPI (200 W)	69.95^d^ ± 0.01	16.59 ^a^ ± 0.08	13.45^a^ ± 0.06
U-OPI (400 W)	71.04^b^ ± 0.08	16.27^b^ ± 0.02	12.68^c^ ± 0.03
U-OPI (600 W)	70.12^c^ ± 0.06	16.68 ^a^ ± 0.02	13.18^b^ ± 0.03

The values are expressed as mean ± standard deviation of triplicate values.

OPI = Oat Protein isolate.

U-OPI = Ultrasound Treated Oat Protein isolate.

At an ultrasonic treatment of 400 W, there was a relative decrease in the α-helix content and an increase in the β-sheet content, indicating an expansion of the protein's secondary structure. This change aligns with the breakdown of the protein's microstructure, which also a reason to contributed to the reduction in the protein's molecular weight. Under the ultrasonic treatment of 600 W, the protein fragments were accumulated locally, resulting in the increase of α-helix content and the decrease of β-sheets content. The reduction in the content of β-sheet along with increase in α-helix and β-turn is indicator of partial denaturation [44] and exposure of reactive and hydrophobic groups which improve the functional properties like, emulsification and solubility. These changes increase the functionality of oat protein isolates in food system [45], [46].

### 3.4 Tertiary structure of oat protein isolates

**Fig. 3:**
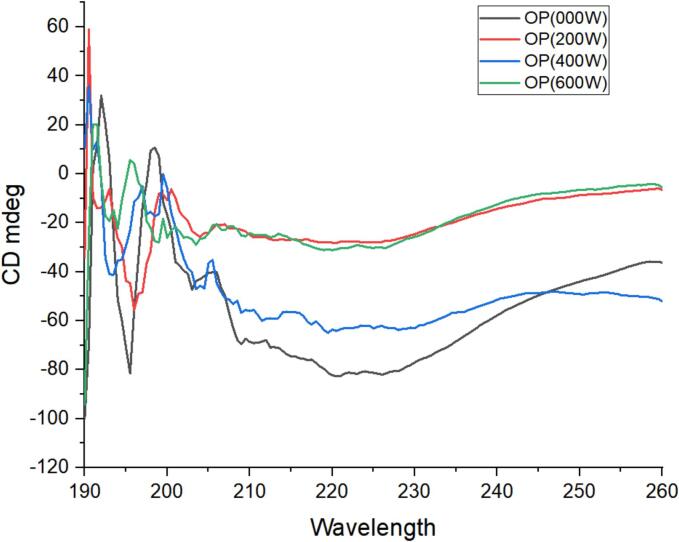
Impact of ultrasound-treatment on Tertiary structure of oat protein isolates (Circular dicorism spectra).

In order to estimate the tertiary structure of protein circular dichroism (CD) was used [47]. In current study, CD spectrum of OPI was used to analyze the conformational variation in tertiary structure of OPI after ultrasonic treated samples (0W, 200W, 400W and 600W) (Fig. 3). In general, a positive band was observed at 190.5 nm in 200 W ultrasonic treated samples, while strong negative band was observed at 196 nm. In 400 W ultrasonic treatment a positive band was observed at 190.5 nm, whereas, strong negative band was observed at 220 nm. In 600 W ultrasonic treated sample a positive band was observed at 190.5 nm and 190 nm while negative band was observed at 260 mm. As shown in Fig. 3. OPI with different ultrasonic wave power treatment showed a great similarity. Like, strong negative peaks at 220 nm with weak shoulder peaks at 215 nm which associated with α-helical structure, with appearance of positive band at 190.5 mm manifests the increase in proportion of β-sheets. There are research-based evidences in recent studies regarding the non-invasive nature of controlled ultrasonic treatment that modulate protein structure and improve its functionality like interaction with biomolecules better digestibility and enhanced enzyme activity [48].

### 3.5 Molecular force of oat protein isolates

**Fig. 4:**
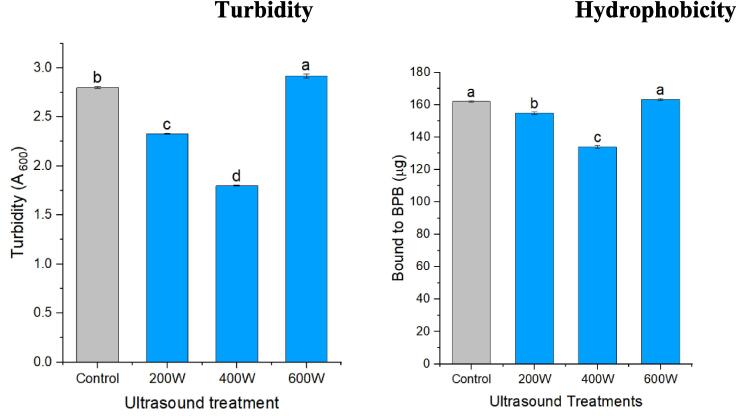
Impact of ultrasound-treatment on turbidity and hydrophobicity of oat protein isolates.

Turbidity of protein decreases significantly under low power ultrasonic treatment (200–400 W), but increases significantly under high power (600 W) ultrasonic treatment, indicating that while moderate ultrasonic treatment enhances protein dissolution, excessive ultrasonic exposure leads to a reduction in solubility (Fig. 4). The reason of this because of the exposure of the hydrophilic regions due to structural alterations and increase amounts of soluble polypeptides during appropriate ultrasonic treatment [38], [43], [49]. Which is consistent with the molecular distribution of proteins, the solvability of low molecular weight (Mw) is higher than that of high molecular weight (Mw) protein polymer. Also, with the change of hydrophobic interaction. moderate ultrasonic treatment destroys hydrophobic groups and thus promotes protein dissolution. On the other hand, excessive ultrasonic treatment significantly destroys protein structure and exposed more hydrophobic groups, which is not favorable to protein dissolution [50].

### 3.6 Amino acid content of oat protein isolates

**Fig. 5:**
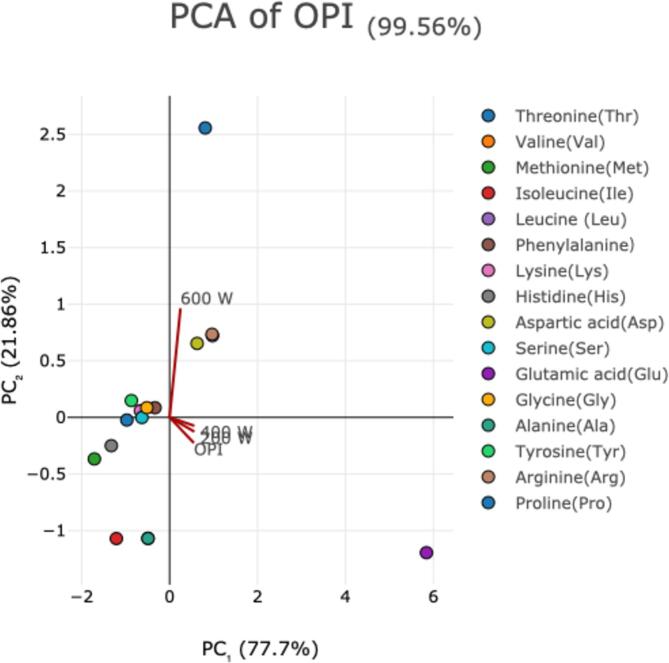
Principal component analysis of ultrasound-treatment on amino acid composition of oat protein isolates.

According to principal component analysis (Fig. 5) ultrasonic treatment reduced the concentration of some of the amino acids (such as Ile, Glu, Gly, etc.), and their contents decreased significantly with the increasing of ultrasonic wave power. Table 3 showed the results of amino acid content. Both of the Total amino acids (TAA) and Essential amino acids (EAA) also showed an overall decreasing trend, suggesting that ultrasonic treatment weakened the nutritional value of oat protein isolates. It is worth noting that although ultrasound destroyed some of the amino acids, the content of Tyr increased with the increase of ultrasonic power, and the content of Pro also increased significantly at high wave power (600 W). This is because that ultrasonic treatment has less damaging effect to these two amino acids, which is favorable to the enrichment of Tyr and Pro.

**Table 3:** Impact of different powers of ultrasound-treatment on amino acid composition of oat protein isolates.

	**Amino acids (mg/100 mg)**	**OPI**	**200 W**	**400 W**	**600 W**	**WHO/FAO standard (adults)**
	Threonine(Thr)	2.76^a^ ± 0.01	2.33^c^ ± 0.02c	2.35^c^ ± 0.04	2.56^b^ ± 0.04	0.9
	Valine(Val)	4.27 ^a^ ± 0.05	3.34 ^a^ ± 0.03	3.79^a^ ± 0.07	0.45^b^ ± 0.03	1.3
	Methionine(Met)	1.35 ^a^ ± 0.03	1.32^a^ ± 0.01	1.32 ^a^ ± 0.01	1.33 ^a^ ± 0.02	1.7
Essential Amino Acid (EAA)	Isoleucine(Ile)	2.92 ^a^ ± 0.05	2.37^b^ ± 0.00	2.31^b^ ± 0.01	0.04^c^ ± 0.00	1.3
	Leucine (Leu)	5.97 ^a^ ± 0.05	5.45^b^ ± 0.01	5.29^c^ ± 0.00	5.38^b^ ± 0.00	1.9
	Phenylalanine）	4.36 ^a^ ± 0.08	3.20^b^ ± 0.01	3.14^b^ ± 0.02	3.22^b^ ± 0.00	1.9
	Lysine(Lys)	3.34 ^a^ ± 0.03	2.83^c^ ± 0.00	2.79^c^ ± 0.02	2.93^b^ ± 0.00	1.6
	Histidine(His)	2.47 ^a^ ± 0.06	1.69^b^ ± 0.05	1.73^b^ ± 0.04	1.86^b^ ± 0.01	1.6
	Aspartic acid(Asp)	5.4 ^a^ ± 0.01	4.81^c^ ± 0.02	4.75^c^ ± 0.04	5.03^b^ ± 0.02	
	Serine(Ser)	3.62 ^a^ ± 0.02	2.83^b^ ± 0.01	2.78^b^ ± 0.03	2.83^b^ ± 0.05	
	Glutamic acid(Glu)	16.1 ^a^ ± 0.50	14.51^b^ ± 0.08	14.39^b^ ± 0.07	3.83^c^ ± 0.02	
	Glycine(Gly)	3.52 ^a^ ± 0.06	3.12^b^ ± 0.01	3.05^b^ ± 0.01	3.07^b^ ± 0.03	
Non-Essential Amino Acid (NEAA)	Alanine(Ala)	4.27 ^a^ ± 0.05	3.34 ^a^ ± 0.03	3.79 ^a^ ± 0.07	0.45^b^ ± 0.03	
	Tyrosine(Tyr)	2.31^c^ ± 0.03	2.63^b^ ± 0.07	2.78^b^ ± 0.08	2.96^a^ ± 0.00	
	Arginine(Arg)	6.19 ^a^ ± 0.04	5.28^c^ ± 0.05	5.19^c^ ± 0.04	5.43^b^ ± 0.00	
	Proline(Pro)	3.58^b^ ± 0.1b	4.26^b^ ± 0.09	5.46^b^ ± 1.60	9.35^a^ ± 0.01	
Essential Amino Acid (EAA)		27.45^a^ ± 0.15	22.56^b^ ± 0.16	22.75^b^ ± 0.82	17.78^c^ ± 0.03	
Total Amino Acid (TAA)		72.04 ^a^ ± 0.3	63.7^b^ ± 0.6b	64.94^b^ ± 2.70	54.26^c^ ± 0.03	

The values are expressed as mean ± standard deviation of triplicate values.

### 3.7 Thermal properties of oat protein isolates

**Fig. 6:**
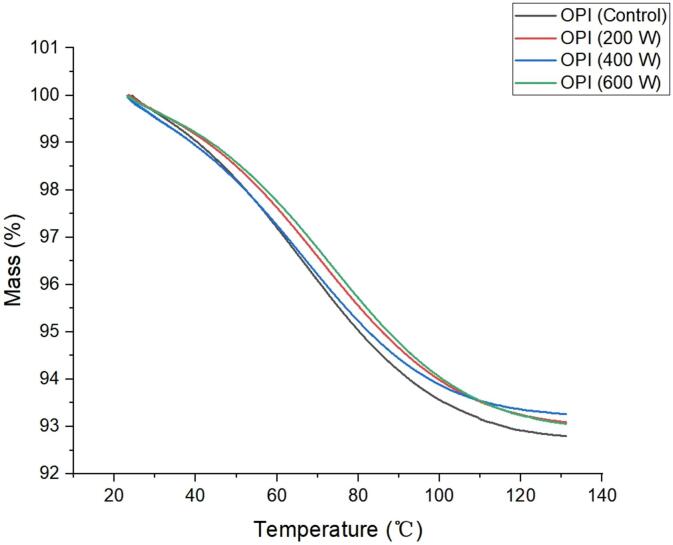
Impact of ultrasound-treatment on thermal behavior of oat protein isolates.

The Thermal properties of oat protein isolates in derivative thermogravimetry curve (DTG) (Fig. 6) showed that the protein of oat was between 30–120 °C. There was no significant difference in the DTG curves of oat proteins extracted by different ultrasonic powers, which indicated that ultrasonic treatment had no effect on the thermochemical properties of the proteins. On the whole, the weight loss rate of the protein in the control group was slightly higher than that of the protein after ultrasonic treatment. This difference may be attributed to the compact structure of natural oat protein which retained more bound water, that is released with protein decomposition at high temperature conditions [49].

The outcomes of current study suggest that thermochemical characteristics of oat protein were not significantly affected as similar pattern of thermal degradation was observed in DTG curves of ultrasonically treated samples in comparison to control samples. The findings of the current works align with the outputs of a previous study where moderate ultrasound intensities improved the structural and functional characteristics, without imparting significant alterations in decomposition behavior and thermal stability [51]. Despite minor variations were observed in thermal behavior of oat protein isolates, its potential impact on functional characteristics like texture, protein aggregation and water retention, play a notable role in quality improvement of protein fortified foods [52], [53].

## 4 Conclusion

The findings of this study indicate that high-intensity ultrasound waves (200 W, 400 W and 600 W) have significantly improved the structural and thermal properties of OPI, while insignificant changes to its amino acid profile was observed. Ultrasonic treatment at 400 W induced the most suitable changes in secondary and tertiary protein structure due to the uncoiling of α-helices and β-sheets, resulting in enhanced overall functionality of the oat protein. Moreover, the number of soluble polypeptides was raised due to structural modifications that also expose hydrophilic regions, increasing the total amino acid and essential amino acid content of OPI, surpassing the FAO and WHO standards. These improvements in the structural and functional characteristics of ultrasonicated oat protein are expected to support the formulation of oat protein-based food products and beverages due to enhanced techno-functional characteristics. Nevertheless, further research is needed to explore other properties including emulsification, foaming capacity, zeta potential, and gelation to ensure the production of stable, protein-rich food products.

### Funding

This work was supported by Shaanxi International Science and Technology Cooperation Bases [2024GH-GHJD-23]; National Oat and Buckwheat Industrial Technology System (CARS-07-E1].

## CRediT authorship contribution statement

**Hamad Rafique:** Conceptualization, Formal analysis, Methodology, Data curation, Writing – original draft. **Pai Peng:** Conceptualization, Formal analysis, Methodology. **Xinzhong Hu:** Supervision,Funding acquisition, Writing – review & editing. **Kanza Saeed:** Investigation, Data curation, Writing – review & editing. **Muhammad Zubair Khalid:** Methodology, Data curation, Writing – review & editing. **Waseem Khalid:** Data curation, Conceptualization, Methodology, Writing – review & editing. **Sonia Morya:** Conceptualization, Methodology, Data curation, Validation, Writing – review & editing. **Tawfiq Alsulami:** Writing – review & editing; Funding acquisition. Data curation. **Robert Mugabi:** Data curation, Writing – review & editing, Validation. **Gulzar Ahmad Nayik:** Data curation, Conceptualization, Validation, Writing – review & editing.

## Declaration of competing interest

The authors declare that they have no known competing financial interests or personal relationships that could have appeared to influence the work reported in this paper.
